# Approaching fast ion transport via anion–dipole interaction in weakly solvated electrolytes enables stable Li-plating chemistry

**DOI:** 10.1093/nsr/nwaf065

**Published:** 2025-02-22

**Authors:** Min Niu, Liwei Dong, Xingyu Chen, Rong-Juan Feng, Qian Li, Hang Qi, Sen Xin, Jia-Yan Liang, Chunhui Yang, Yu-Guo Guo

**Affiliations:** State Key Laboratory of Space Power-Sources, School of Chemistry and Chemical Engineering, Harbin Institute of Technology, Harbin 150001, China; State Key Laboratory of Space Power-Sources, School of Chemistry and Chemical Engineering, Harbin Institute of Technology, Harbin 150001, China; State Key Laboratory of Space Power-Sources, School of Chemistry and Chemical Engineering, Harbin Institute of Technology, Harbin 150001, China; CAS Key Laboratory of Molecular Nanostructure and Nanotechnology, CAS Research/Education Center for Excellence in Molecular Sciences, Beijing National Laboratory for Molecular Sciences, Institute of Chemistry, Chinese Academy of Sciences, Beijing 100190, China; CAS Key Laboratory of Molecular Nanostructure and Nanotechnology, CAS Research/Education Center for Excellence in Molecular Sciences, Beijing National Laboratory for Molecular Sciences, Institute of Chemistry, Chinese Academy of Sciences, Beijing 100190, China; State Key Laboratory of Space Power-Sources, School of Chemistry and Chemical Engineering, Harbin Institute of Technology, Harbin 150001, China; CAS Key Laboratory of Molecular Nanostructure and Nanotechnology, CAS Research/Education Center for Excellence in Molecular Sciences, Beijing National Laboratory for Molecular Sciences, Institute of Chemistry, Chinese Academy of Sciences, Beijing 100190, China; School of Chemical Sciences, University of Chinese Academy of Sciences, Beijing 100049, China; State Key Laboratory of Space Power-Sources, School of Chemistry and Chemical Engineering, Harbin Institute of Technology, Harbin 150001, China; CAS Key Laboratory of Molecular Nanostructure and Nanotechnology, CAS Research/Education Center for Excellence in Molecular Sciences, Beijing National Laboratory for Molecular Sciences, Institute of Chemistry, Chinese Academy of Sciences, Beijing 100190, China; State Key Laboratory of Space Power-Sources, School of Chemistry and Chemical Engineering, Harbin Institute of Technology, Harbin 150001, China; CAS Key Laboratory of Molecular Nanostructure and Nanotechnology, CAS Research/Education Center for Excellence in Molecular Sciences, Beijing National Laboratory for Molecular Sciences, Institute of Chemistry, Chinese Academy of Sciences, Beijing 100190, China; School of Chemical Sciences, University of Chinese Academy of Sciences, Beijing 100049, China

**Keywords:** Li-based battery, interfacial stability, weakly solvated electrolytes, ion-transport kinetics, anion–dipole interactions

## Abstract

The graphite/Li-metal hybrid anode demonstrates great potential in cycling stability and energy density with designed weakly solvated electrolytes when considering the common issue of solvent co-intercalation and vulnerable interface chemistry with a graphite anode and Li anode, respectively. The weakly solvated electrolytes show weak ion–dipole interaction and promote rapid desolvation but are faced with sluggish ion-transport kinetics, thus inducing high overpotential and Li-dendrite formation. Herein, by applying methyl propionate as a weakly coordinated cosolvent, a loose solvation shell that is regulated by anion–solvent interaction enables weakened Li^+^–anion interaction while maintaining adequate anion participation, featuring a facilitated bulk ion-transport route via anion dissociation, originally achieving a high ionic conductivity of 17.74 mS cm^−1^ in weakly solvated electrolytes at 25°C. Consequently, this advanced electrolyte design markedly mitigates concentration polarization and regulates uniform Li deposition, and thus the hybrid anode achieves 99.8% average coulombic efficiency within 1500 cycles at 4C and improved cycling stability at a low N/P ratio of 0.5, making a breakthrough in alkali-metal-ion batteries.

## Teaser

Breaking the dilemma of desolvation and ion-transport kinetics in weakly solvated electrolytes for a dendrite-free Li-based anode.

## INTRODUCTION

**Figure 1.**
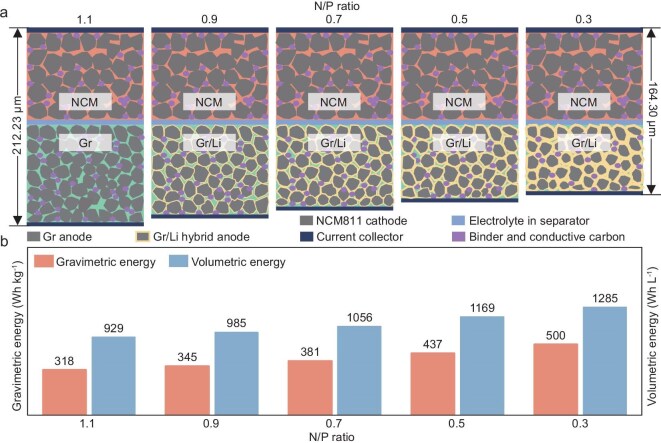
Schematic illustrating the correlation of (a) the stacking thickness of the cell and (b) the energy density with the decreasing N/P ratio in the Gr/Li hybrid anode study.

Graphitic anodes have functioned as the prevailing Li-ion host structure for energy storage over the past three decades, primarily owing to their remarkable stability and prolonged cycling lifespan. However, sole reliance on the Li-ion intercalation chemistry with a capacity limit (LiC_6_,372 mAh g^−1^) in graphite-anode-based Li-ion batteries (LIBs) fails to meet the urgent demands for higher energy density in various emerging fields. In this view, the collocation of the Li-metal anode (LMA) and high-nickel-layered oxide cathode (e.g. LiNi_0.8_Co_0.1_Mn_0.1_O_2_ (NCM811)) appear to be the preferred choice for the realization of high-energy batteries of 500 Wh kg^−1^ and 1000 Wh L^−1^ [1–3]. Nevertheless, the inferior interfacial stability that is caused by dead Li and the intractable degradation of the solid–electrolyte interphase (SEI) shorten the cycling lifespan and constrain the practical application of LMAs [4–9]. Interestingly, a novel graphite/Li-metal hybrid anode (Gr/Li), which inclines the partial Li ion to be plated reversibly on the fully lithiated graphite surface for achieving capacity compensation (Fig. 1a), has shown thermodynamic feasibility until the potential becomes negative enough, referring to Li^+^/Li. The Gr/Li anode in batteries permits a notable decrease in the proportion of anodic graphite, thereby enabling a refined trade-off between energy density and cycling lifespan. Therefore, high specific energy can be realized in a LIB with an uncommonly low negative/positive (N/P) area capacity ratio of <1.1 [10–12]. Attributed to the decreased electrode thickness and weight, batteries employed in this Gr/Li hybrid anode demonstrate a theoretical energy density of nearly 450 Wh kg^−1^ at an N/P ratio of 0.5 and 500 Wh kg^−1^ at an N/P ratio of 0.3, as shown in Fig. 1b and Supplementary Note S1.

Unfortunately, the commercial ethylene carbonate (EC)-based electrolytes do not fit with Gr/Li hybrid anode systems, due to their strong dipole force and the high reduction potential of the Li^+^–EC pair. These inherent properties induce the poor Li^+^ desolvation kinetics at the interface and the increased diffusion barrier of Li^+^ through a thick organics-enriched SEI owing to the dramatic side reactions between the Li metal and the solvents [13–16]. In this regard, weakly solvated electrolytes that are made by replacing or diluting the EC with a polyfluorinated solvent, including fluoroethylene carbonate (FEC), hydrofluoroethers (HFEs), etc., has been verified as a successful deployment in Gr and LMA, which can effectively reduce the interaction between Li^+^ with solvents and allow more of the anion to participate into the Li^+^ primary solvation shell (PSS) [5,17,18]. As a result, these electrolytes facilitate desolvation and derive an inorganics-enriched SEI via anion reduction, favoring reversible Li plating/stripping with high coulombic efficiency (CE). Despite the significant development of fluorinated carbonate-based electrolytes, the ionic conductivity of all these electrolytes is largely reduced after the fluorination, leading to inferior ion-transport kinetics in terms of severe concentration polarization and high overpotential, especially under high-rate or high-areal-loading conditions. Note that the homogenized Li-ion flux through the inorganics-enriched SEI is required for guiding the uniform Li-metal plating [19], while sluggish ion transport further induces the generation of Li dendrite or ‘dead Li’ and even thermal runaway. Therefore, the ideal electrolyte requires contradictory properties of high desolvation kinetics but high ionic conductivity and an inorganics-enriched interphase, which are hardly ever achieved in common weakly solvated electrolytes simultaneously. Moreover, considering the high sensitivity of ion-transport kinetics and the interfacial reaction to the subtle interactions within the cation solvents–anion complexes, it is necessary to uncover the mechanism of how the intrinsic coordination behavior of the ion–dipole acts on the solvation structure and ion conductivity to obtain stable high-energy-density Li-storage electrochemistry.

In this work, we report the strategic regulation of the competitive coordination of the ion–dipole and the cation–anion through the introduction of weakly solvated methyl propionate (MP) into the commercial EC-based electrolyte, thereby enhancing the desolvation kinetics, accelerating Li^+^ transport and achieving a high CE of Li^+^ plating/stripping without Li-dendrite formation for the Gr/Li hybrid anode. The anion–dipole interaction of EC–PF_6_^−^ that is induced by the MP cosolvent favors a loose solvation sheath with less solvent but more anion participation [20–22], in which Li^+^ moves with its flexible Li^+^–EC pair that results from the weak ion–anion interaction and low EC ligancy but strong ion–dipole interaction, endowing the electrolyte with high ionic conductivity (17.74 mS cm^−1^, 25°C) and Li^+^ transference number (0.40). Hence, these properties possess the rapid bulk ion-transport kinetics for dendrite-free Li plating on the Gr surface, ensuring reversible cycling of the Gr/Li hybrid anode, as evidenced by the average CE of 99.8% within 1500 cycles at 4C and the stable cycling lifespan that is extended to 120 cycles with an average CE of 98.6% under the high anodic areal capacity of 3.0 mAh cm^−2^. This work elucidates the fundamental relationship between the PSS and the ion–dipole interaction, and its favorable effect on rapid ion transport and mitigating the concentration polarization for homogeneous Li deposition, thereby enabling efficient Li utilization and stable Li-storage electrochemistry in the Gr/Li hybrid anode for high-energy-density LIBs.

## RESULTS AND DISCUSSION

### Anion–solvent interaction mediating electrolyte solvation structure features

**Figure 2.**
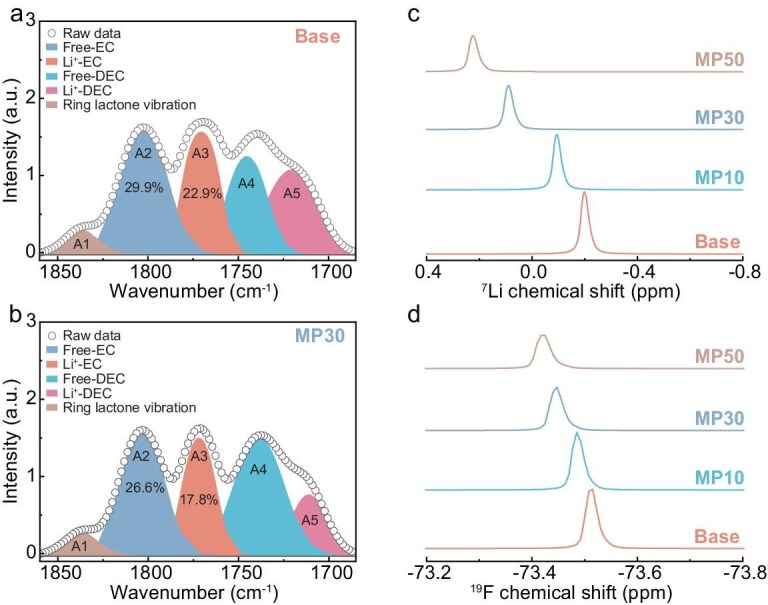
Fitting peaks of the C=O regions of the FTIR spectra for EC and DEC solvents of (a) Base and (b) MP30 electrolytes. (c) ^7^Li NMR and (d) ^19^F NMR spectra of different electrolytes.

To investigate the effect of the anion–dipole interaction of EC–PF_6_^−^ that is induced by MP cosolvent on the PSS structure, ionic transport kinetics and battery performance, four electrolytes are investigated in this study: 1.0 M LiPF_6_ in EC and diethyl carbonate (DEC) (1:1 by volume, denoted as the Base electrolyte), adding varied contents of MP cosolvent into the Base electrolyte additionally for a series of electrolytes (10%, 30% and 50% volume of the Base electrolyte, labeled as MP10, MP30 and MP50 electrolytes, respectively). First, the Li‖Cu cell with the MP30 electrolyte demonstrates a moderate Li nucleation overpotential (121.2 mV) and a maximal Li stripping capacity (4.56 mAh cm^−2^) (Fig. S1), signifying a marked enhancement in the reversibility of deposited Li in the MP30 electrolyte. Fourier transform infrared (FTIR) spectra were conducted to reveal the evolution of the electrolyte solvation microstructures that were induced by the MP cosolvent based on the high sensitivity of the C=O group in the EC/DEC molecules (Fig. S2) [23,24]. As observed in Fig. 2a and b, the peak of the C=O group in Li^+^–EC red-shifts from 1769.7 cm^−1^ in the Base electrolyte to 1771.9 cm^−1^ in the MP30 electrolyte with reduced stretching vibration energy as well as the peaks of the C=O group in Li^+^–DEC (blue-shifts from 1714.4 to 1711.7 cm^−1^) (Table S1), pointing to the reduced Li^+^–solvents pairs by adding MP cosolvent, which induces an enhanced ion–dipole interaction and allows more PF_6_^−^ to integrate into the Li^+^ PSS. This lower proportion of EC/DEC coordinating with Li^+^ is also quantified by deconvoluting the FTIR spectrum by using the Voigt function, demonstrating a decreased area percentage from 22.9% of Li^+^–EC pairs in the Base electrolyte to 17.8% in the MP30 electrolyte, which corroborates the weakening of solvation in the MP30 electrolyte (Table S1). In contrast, the variation in the characteristic MP peaks is negligible in the MP30 electrolyte (Fig. S3), suggesting weaker competitive coordination to Li^+^ than that of EC/DEC. Besides, it is found that the Li^+^–PF_6_^−^ pair ratio increases in the MP30 electrolyte (Fig. S4), deriving from the weak solvating ability with a lower dielectric constant of MP (i.e. *e*_MP_ = 6.2) than that of EC (i.e. *e*_EC_ = 89.1), giving rise to the more PF_6_^−^ participation in the PSS [25–27]. Therefore, there is low EC/DEC ligancy as the MP exists, while PF_6_^−^ can contact Li^+^ at a high frequency, demonstrating the construction of an anion-rich solvation structure in the MP30 electrolyte. In conclusion, the MP cosolvent diminishes the solvating power of the EC/DEC, thereby facilitating Li^+^ detachment from the solvent—a crucial aspect for enabling rapid interfacial ion transport and suppressed electrolyte decomposition near the Gr anode.

The intrinsic evolution of the Li^+^ binding environment is further characterized by using nuclear magnetic resonance (NMR) spectra [28]. As shown in Fig. S5, the varied downfield shifts are observed in the ^1^H NMR spectra for –CH_3_ in the EC molecule after MP introduction due to the weakened solvation power, demonstrating the decreased shielding effect of Li^+^ for the EC solvent but maintaining a strong Li^+^–EC interaction. The ^7^Li peak position gradually shifts downfield with increasing MP amounts from 10% to 50% (Fig. 2c), indicating a reduction in the electron density around the Li^+^ caused by the attenuated degree of solvent that is participating in the solvation sheath. Usually, the deficiency of the solvent molecules may allow the PF_6_^−^ to stay closer to the Li^+^, thus showing an increased shielding effect of the Li atom and the upfield displacement. However, the ^19^F NMR spectrum also undergoes a downfield with enhanced MP content; it is plausible that the potential PF_6_^−^–solvent interaction can dispel electron-dense PF_6_^−^ near the Li^+^ and cause an enhanced deshielding effect as more PF_6_^−^ occupies the inner solvation sheath with the addition of the MP cosolvent (Fig. 2d), demonstrating the existence of the anion–dipole interaction of PF_6_^−^–EC, thus leading to a weakened Li^+^–PF_6_^−^ interaction. Consequently, the competitive affinity with Li^+^ between the decreased Li^+^–EC pairs with enhanced cation–dipole interaction and the more Li^+^–PF_6_^−^ pairs with weakened cation–anion interaction in the MP30 electrolyte leads to a loose Li^+^ coordination environment, which further leads to the formation of an anion-derived SEI and fast desolvation kinetics to improve the cycling stability of the Gr/Li anode.

**Figure 3.**
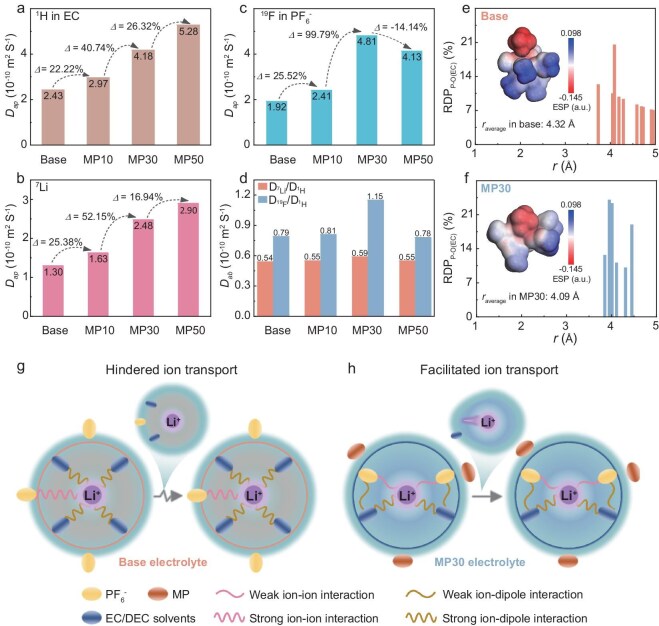
The *D*_ap_ of (a) EC, (b) Li^+^ and (c) PF_6_^−^ in electrolytes with various MP contents monitored by the ^1^H, ^7^Li and ^19^F signals by using the DOSY–NMR spectrum, respectively. (d) The *D*_ab_ of Li^+^ and PF_6_^−^ in electrolytes with various MP contents monitored by the ^7^Li/^1^H and ^19^F/^1^H by using the DOSY–NMR spectrum. The RDP of P–O(EC) in (e) Base and (f) MP30 electrolytes; the insets show the ESP of the Li^+^ solvation structures. Schematic diagram of the interaction between Li^+^, anions and solvents in the solvation structure of (g) Base and (h) MP30 electrolytes.

NMR diffusion order spectroscopy (DOSY) was employed to probe Li-ion (^7^Li for Li^+^), solvent (^1^H for –CH_3_ of EC) and anion (^19^F for PF_6_^−^) self-diffusion in electrolytes (Fig. S6) [29,30]. Figure 3a–c shows the apparent diffusion coefficient (*D*_ap_) of components within the electrolytes with gradient MP contents. With the content of MP increasing, the *D*_ap_ of the EC solvent demonstrates a consecutive increase from 2.43 × 10^−10^ m^2^ S^−1^ in the Base electrolyte to 5.28 × 10^−10^ m^2^ S^−1^ in the MP50 electrolyte, as well as a *D*_ap_ of Li^+^ from 1.30 × 10^−10^ m^2^ S^−1^ in the Base electrolyte to 2.90 × 10^−10^ m^2^ S^−1^ in the MP50 electrolyte, which is partly attributed to the decreased viscosity within weakly solvated electrolytes (from 5.54 cP of the Base electrolyte to 4.48 cP of the MP30 electrolyte at 25°C) (Table S2). Besides, the weakened coordination of the Li^+^ with the solvents might lead to a smaller solvation cluster, thus allowing fast solvent transport carrying the Li^+^ together despite the anion disturbance. It is notable that, as the MP content exceeds 30%, the *D*_ap_ of the PF_6_^−^ shows a distinct decline compared with the EC and Li^+^, decreasing from 4.81 × 10^−10^ m^2^ S^−1^ in the MP30 electrolyte to 4.13 × 10^−10^ m^2^ S^−1^ in the MP50 electrolyte. The results further demonstrate the existence of the competitive coordination between the solvent and PF_6_^−^ with Li^+^, explaining the dragging effect of the excessively coordinated PF_6_^−^ on Li^+^ diffusivity. The absolute diffusivity (*D*_ad_) is defined by dividing the *D*_ap_ of the ion by the *D*_ap_ of the EC, eliminating the viscosity effect of the electrolytes. As shown in Fig. 3d, the *D*_ad_ of Li^+^ with respect to the EC remains almost constant. In a sharp contrast, as MP is gradually added, the *D*_ad_ of the PF_6_^−^ with respect to the EC exhibits an extreme point. Specifically, in the MP30 electrolyte, the *D*_ad_ of the PF_6_^−^ reaches its maximum value of 1.15 × 10^−10^ m^2^ S^−1^. At this point, the competitive interaction between the PF_6_^−^ and the EC coordinating with Li^+^ reaches an ultimate state, resulting in the lowest constraint by the anion on the diffusion of Li^+^, contributing to the increase in free Li^+^ and high Li^+^ transference number. As the MP content increases to 50%, there is a pronouncedly competitive affinity to Li^+^ of the PF_6_^−^ compared with the EC, with the anions being strongly bound by Li^+^, resulting in a decreased *D*_ad_ of the PF_6_^−^. This change may expand the probable Li^+^ conduction route via anion dissociation, which regulates the uniform Li deposition and achieves superior cycling stability of the Gr/Li hybrid anode.

To further validate the different interactions between the anion and the solvents (e.g. EC), the P–O (EC) radial distribution probability (RDP) is replicated by using molecular dynamic simulation. For the MP30 electrolyte, the average radius (*r*_average_) between the PF_6_^−^–O(EC) species is 4.09 Å, which is lower than that in the Base electrolyte (4.32 Å), demonstrating that the MP existence reinforces anion–solvent interactions, as shown in Fig. 3e and f. The geometrically optimized PSSs of the Base and MP30 electrolytes are shown in Fig. S7. The anion–dipole interaction between PF_6_^−^ and the EC induces the polarization of the electron cloud in the PF_6_^−^ toward the center oxygen in the EC molecules, thus diminishing the electronegativity of F and enhancing the ion–dipole interaction of Li^+^–EC. Notably, the distribution of negative charge in the MP30 electrolyte is more scattered, as displayed by the electrostatic potential (ESP) distribution in the insets of Fig. 3e and f. Consequently, based on the preceding analysis for the Base and MP30 electrolytes, we establish the schematic diagram of the Li^+^ solvation structure in two electrolytes, containing several key interactional features of the Li^+^–PF_6_^−^, Li^+^–solvent and PF_6_^−^–solvent, as shown in Fig. 3g and h. For the Base electrolyte, the strong solvating power of EC/DEC results in significant incorporation of EC/DEC into the first solvation shell, causing the PF_6_^−^ to be excluded from the first solvation shell and solvent-dominated solvation structure, featuring the strong Li^+^–solvent interaction and the sluggish ion transport carrying the bulky solvation cluster. During the electrochemical cycling, Li^+^ encounters difficulty in overcoming the constraints imposed by the EC/DEC molecules, which leads to sluggish ion transport and desolvation kinetics, provoking organics-enriched SEI, parasitic reaction and even Li dendrite at the Gr/Li hybrid anode surface. Conversely, the presence of MP attenuates the Li⁺–solvent interactions, facilitating the anions to enter the solvation shell and inducing the anion–solvent interaction. This means the formation of a loose coordination structure between the solvent and anions with Li⁺, which achieves adequate freedom of Li^+^ movement and facilitates rapid bulk ion migration with less but strong solvent coordination. These analyses unveil the regulatory mechanism of anion–solvent interactions on the ion-transport kinetics of the bulk electrolyte and electrode/electrolyte interface.

### Bulk ion-transport features

**Figure 4.**
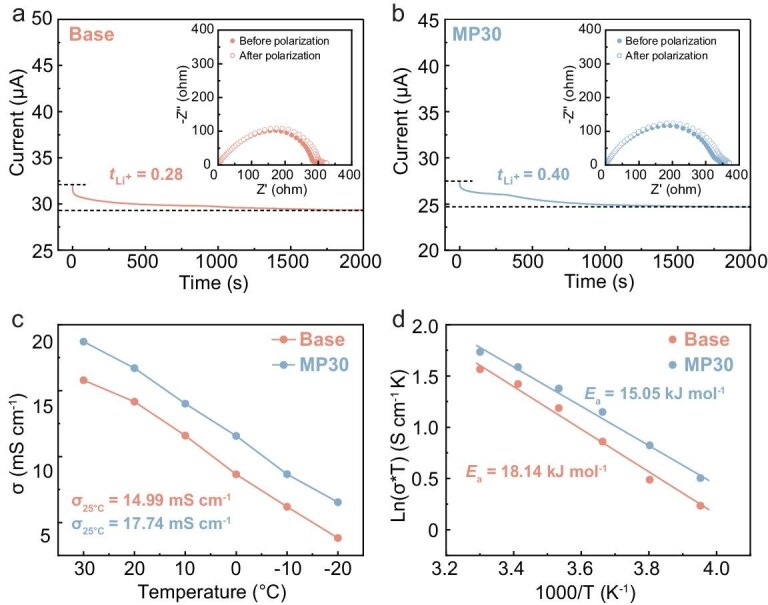
Li^+^ transference number of (a) Base and (b) MP30 electrolytes; the inset shows the Nyquist impedance before and after polarization. (c) Ionic conductivity and (d) kinetic energy barrier of the bulk charge transfer by Arrhenius analysis in Base and MP30 electrolytes.

The impact of the solvation structure is further explored in relation to the thermodynamics of Li^+^ transport and charge transfer in the bulk electrolyte. Benefitting from the Li^+^–EC pair that diffuses flexibly with the least constraint by the PF_6_^−^ in a loose solvation shell that is regulated by the MP cosolvent, the MP30 electrolyte enables the improved bulk Li^+^ transport kinetics, realizing a much higher Li^+^ transference number of 0.40 than that of the Base electrolyte (0.28), favoring reduced polarization during the fast-charging process, as depicted in Fig. 4a and b. In addition, the charge-transfer resistance (*R*_ct_) of symmetrical Stee‖Steel cells is measured through electrochemical impedance spectroscopy (EIS) at varied temperatures (Fig. S8) [31]. The cells with the MP30 electrolyte at all temperatures achieve substantially lower charge-transfer impedance compared with those with the Base electrolyte, suggesting that the presence of the MP cosolvent greatly promotes the bulk charge-transfer behavior. As a result, the MP30 electrolyte demonstrates a higher ionic conductivity (*σ*) over a wide temperature range from +30 to –20°C than the Base electrolyte (e.g. σ_MP30_ = 17.74 mS cm^−1^ and σ_Base_ = 14.82 mS cm^−1^, 25°C) because of the stronger Li^+^–EC interaction despite maintaining lower solvent participation, which is advantageous for facilitating rapid bulk Li^+^ transport (Fig. 4c). The activation energy (*E*_a_) of the bulk Li^+^ transport is further calculated from temperature-dependent EIS by using an Arrhenius equation. As shown in Fig. 4d, the MP30 electrolyte has the lower activation energy for the bulk Li^+^ transport, confirming rapid bulk transport kinetics. The above results demonstrate that our strategy of regulating the competitive coordination of Li^+^ by the regulated interaction between the Li^+^, PF_6_^−^ and solvent molecules has successfully optimized the solvation structure to promote ion transport and facilitate electrochemical kinetics. It is predicted that the fast Li^+^ transfer kinetics favor uniform Li^+^ flux distribution, thus adjusting the homogeneous Li deposition on the Gr surface contrasts with the tip-growing deposition in the Base electrolyte (Fig. S9).

### Effect of loose solvation shell induced by MP on electrochemistry stability

**Figure 5.**
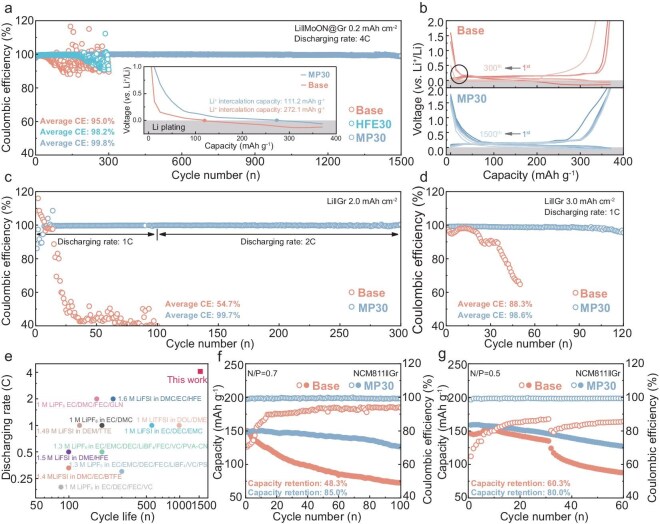
(a) Cycling performance and (b) voltage profiles of the Li‖MoON@Gr cells using different electrolytes with a Gr area capacity of 0.2 mAh cm^−2^, discharging at 4C. The inset in (a) shows the Li^+^ intercalation capacity at the first cycle. The black-circled region in (b) exhibits polarization and overpotential as the cycle deteriorates. (c) The cycling performance of the Li‖Gr cells using different electrolytes with Gr area capacity of 2.0 mAh cm^−2^, discharging at 1C/2C. (d) The cycling performance of the Li‖Gr cells using different electrolytes with Gr area capacity of 3.0 mAh cm^−2^, discharging at 1C. (e) Comparison of the discharge rate and the corresponding cycle life of this work and recent relevant reports for electrolytes applied in the Gr/Li hybrid anode (detailed information is summarized in Table S3). Cycling performance of the NCM811‖Gr full cells using different electrolytes at N/P ratios of (f) 0.7 and (g) 0.5.

Typically, Li-metal plating becomes kinetically feasible, substituting insertion into Gr layers once the discharging voltage drops below 0 V vs. Li^+^/Li. To assess the stability of mixed-mode lithium storage in the Gr anode, a steady lithiated capacity assessment involving Li^+^ intercalation and Li^+^ plating (Fig. S10) is conducted on the Li‖Gr cells in different electrolytes at varying current densities and area capacities. A novel MoO*_x_*–MoN*_x_* layer (MoON) that is integrated onto the Gr surface has been tested recently in our research to facilitate a stable Gr–electrolyte interface that is characterized by the promoted desolvation, accelerated Li^+^ migration and suppressed interfacial solvent reduction [32], which is chosen as the anode for hybrid Li-storage assessment. Moreover, the HFE-added electrolyte (HFE30), distinguished by its reduced Li^+^–solvent interaction and lacking ion–dipole interaction in the Li^+^ PSS, thus favoring the facilitated desolvation but sacrificing the ionic conductivity [25,33,34], is also utilized as a control. Initially, the Li‖MoON@Gr cells with an anodic area capacity of 0.2 mAh cm^−2^ (based on the mass of the Gr active material) are assembled with various electrolytes to evaluate the hybrid Li-storage behavior at a fast discharging rate of 4C. As shown in the inset in Fig. 5a, the Li‖MoON@Gr cell with the Base electrolyte suffers a rapid voltage drop, providing a Li^+^ intercalation capacity of only 111.2 mAh g^−1^ compared with that of 272.1 mAh g^−1^ in the MP30 electrolyte in the first cycle, suggesting the favorable reaction kinetic with the fast bulk ion transport that is regulated by the lower solvent ligancy with the MP cosolvent introduction. Moreover, the Li‖MoON@Gr cell with the MP30 electrolyte shows exceptional cycling stability, with an average CE within 1500 cycles of 99.8% (Fig. 5a) and a retained charging capacity of 366.8 mAh g^−1^, including a capacity compensation of 218.4 mAh g^−1^ that originates from the highly reversible deposited Li metal at the 1500th cycle (Fig. S11a). While the cells with HFE30 and Base electrolytes show a rapid decline in CE after 180 and 30 cycles, respectively, which is attributed to the inferior bulk ion-transport kinetics, even the disrupted interfacial Li^+^ desolvation kinetics as the cycling proceeds leads to increased concentration polarization and obvious Li^+^ nucleation overpotential (Fig. 5b and Fig. S11b). Under harsher conditions of high areal capacity (Fig. S12), although the cycle stability of the Li‖Gr cells with the HFE30 electrolyte has been extended, it is still quite unsatisfactory. These results emphasize the crucial factor of bulk Li^+^ transport for stable Li storage in the form of the hybrid Li^+^ intercalation and plating simultaneously for the Gr/Li hybrid anode, even when employing the advanced solid–liquid interface design that is achieved by the MoON layer, cooperating with a weakened solvent structure for allowing enhanced interfacial ion transport.

Besides, the developed MP30 electrolyte coupling with the higher Gr mass loading also yielded excellent electrochemical performance. As displayed in Fig. 5C and Fig. S13, the Li‖Gr cell with 2.0-mAh cm^−2^ anodic area capacities using the MP30 electrolyte retains a high average CE of 99.7% and a charging capacity of 371.55 mAh g^−1^ after 300 cycles of discharging at 1C/2C, possessing the Li^+^ intercalation capacity of 69.9 mAh g^−1^ and Li^+^ plating capacity of 300.1 mAh g^−1^ at the 300th cycle. In contrast, the cell with the Base electrolyte has already failed within the first 100 cycles of discharging at 1C. A similarly extended cycling lifespan during 120 cycles is also achieved under the higher anodic area capacity of 3.0 mAh cm^−2^ at a discharging rate of 1C (Fig. 5d and Fig. S14). As summarized in Fig. 5e and Table S3, the Gr/Li hybrid anode with the developed MP30 electrolyte outperforms most of prevalent electrolytes in fast-charging capability and cyclic life [10,35–41]. Moreover, the facilitated bulk ion-transport coupling with rapid desolvation kinetics in the MP30 electrolyte that features a low freezing point (–58.5°C) and high ion conductivity in a wide temperature range also enables stable LMA even at low temperatures, supporting that the NCM811‖Li cell shows excellent cycling stability with 81.4% capacity retention after 50 cycles at –20°C (Figs S15 and S16). To assess the feasibility of the concept that the MP30 electrolyte enhances energy density by modulating reversible Li^+^ plating at the Gr anode, the NCM811‖Gr full cells are assembled. Initially, the NCM811‖Gr cell with the normal N/P ratio of 1.1 using the MP30 electrolyte shows a capacity retention of 87.4% after 200 cycles (Fig. S17a), demonstrating the superior cycling stability of the designed MP30 electrolyte in conventional battery configurations compared with the commercial EC-based electrolyte in this research. Moreover, the NCM811‖Gr cells with N/P ratios of <1.1 exhibit initial capacities that approach that of a cell with an N/P ratio of 1.1 that is cycled from 2.75 to 4.25 V at 0.2C (Fig. 5f and g, and Fig. S17b), surpassing the maximum capacity provided by the Li^+^ intercalation into the limited Gr materials. This suggests the additional storage of Li^+^ in the form of Li metal on the Gr anode during charging. Impressively, the NCM811‖Gr cell with the MP30 electrolyte realizes low-anode-loading stable cycling of 85.0% capacity retention after 100 cycles at an N/P ratio of 0.7 and 80.0% capacity retention after 60 cycles at an N/P ratio of 0.5, respectively, for achieving high Li^+^ utilization efficiency that is manifested by the remaining discharging capacities of 126.2 and 126.9 mAh g^−1^, respectively, as displayed in Fig. 5f and g. In comparison, the cells with the Base electrolyte experience rapid capacity decay and undergo lower and gradually increased CE at the beginning of charge and discharge, which is related to the irreversible active Li consumption transferring to dendritic-Li formation and interphase degradation. These results demonstrate the feasibility and practicality of achieving a high-energy-density battery system that is close to 450 Wh kg^−1^ by employing the proposed MP30 electrolyte matching with the Gr/Li hybrid anode.

### Interfacial failure and thermal stability features

**Figure 6.**
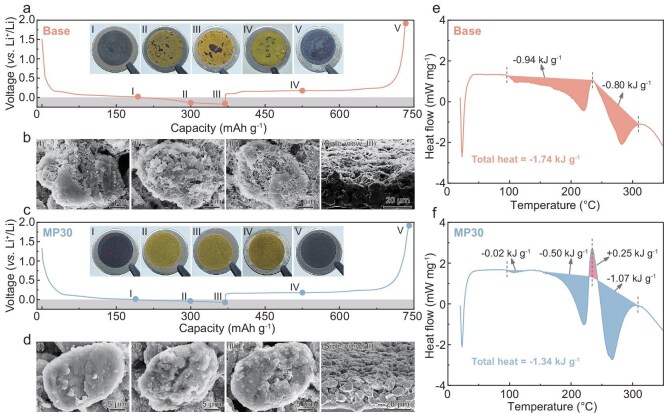
Voltage profiles of Li‖Gr cells cycled for the 30th cycle at 2C in the Base electrolyte (a), and the insets in (a) show optical images of the anodes recorded at five different stages (I–V) marked in the voltage profiles. The top and side-view SEM images (b) of the Gr anodes cycled in the Base electrolyte at the selected stages (I–III). The voltage profiles of Li‖Gr cells cycled for the 30th cycle at 2C in the MP30 electrolyte (c), and the insets in (c) show optical images of anodes recorded at five different stages (I–V) marked in the voltage profiles. The top and side-view SEM images (d) of the Gr anodes cycled in the MP30 electrolyte at the selected stages (I–III). DSC profiles of the Gr/Li hybrid anode with (e) Base and (f) MP30 electrolytes.

The digital photos and scanning electron microscopy (SEM) analysis further elucidate the direct correlation between the morphologies of electrodeposited Li metal and electrochemical performance. As shown in Fig. 6a, as the discharging proceeds to point III (discharged to 370 mAh g^−1^) in the Base electrolyte, the inset digital photos of the electrodes reveal that the dead Li sporadically spread and become more apparent on the yellowish Gr surface. Even when charged up to 2.0 V vs. Li^+^/Li (Fig. 6a, point V), the distribution of dead Li can still be distinctly distinguished by its gray color. As expected, for the Gr cycled in the Base electrolyte, the localized needle-like Li dendrites are observed initially and stack gradually on the cracked surface of the Gr until they envelop almost the entire electrode surface upon reaching a maximum lithiation capacity of 370 mAh g^−1^, featuring massive vast gaps between dendrites that reveal a loose and porous structure (Fig. 6b and Fig. S18a). Such structural degradation and dendritic-Li formation that result from inferior interfacial and bulk ion transfer kinetics lead to poorer cycling performance and lower CE. By contrast, for the Gr electrode that is cycled in the MP30 electrolyte, the surface changes from black to yellowish and ends up with a black surface without any dead Li during the discharging and charging process (Fig. 6c). The dendrite-free Li plating on the Gr surface in the MP30 electrolyte can be attributed to the rapid desolvation and facilitated bulk ion-transport route, resulting in Li metal that is uniformly spread over the lithiated Gr matrix surface (Fig. 6d and Fig. S18b), therefore leading to high ionic utilization and the extended cycling life of the Gr/Li hybrid anode. Furthermore, the SEI that is induced by the anion-rich solvation structure is also beneficial for long-term stable cycling performance. The interfacial chemical reaction process is examined by using X-ray photoelectron spectroscopy (XPS) techniques. As expected, the dendritic lithium with the high chemical reactivity results in drastic electrolyte reduction at the Gr/electrolyte interface during cycling in the Base electrolyte, characterized by the strengthened intensities of the carbon-oxygen-containing species at the Gr surface that gradually dominate the chemical composition of the SEI (Fig. S19a and b). Meanwhile, the XPS of P 2p reveals a high Li*_x_*PO*_y_*F*_z_* ratio of 71.31% at the initial third cycle in the MP30 electrolyte, confirming the anion-derived SEI that is driven by the involvement in the Li^+^ solvation as evidenced by the NMR data (Fig. 2d), and maintains a high proportion throughout the subsequent cycles with lesser solvent decomposition contributing to SEI reconstruction (Fig. S19c and d). In addition, the XPS of Li 1s has an LiF ratio of 36.56 % after cycling in the MP30 electrolyte, which is considerably higher than the 14.20% of the cycled Gr/Li hybrid anode in the Base electrolyte (Fig. S19e and f), proving the regulated LiF-based SEI layer for enhanced interfacial stability, thus supporting the PF_6_^−^-enriched solvation structure with the introduction of the MP. This suggests that the anion-derived inorganics-enriched SEI effectively suppresses lithium dendrite formation, thereby favoring a stable electrode/electrolyte interphase and enhancing interfacial reaction kinetics.

Differential scanning calorimeter (DSC) measurement is performed on the discharged Gr/Li anodes with various electrolytes to evaluate their thermal stability. As shown in Fig. 6e, the total exothermic heat that is generated from the lithiated Gr/Li anode with the Base electrolyte is 1.74 kJ g^−1^, which is considered the catalyst for a succession of exothermic reactions that lead to uncontrollable battery thermal runaway. Interestingly, a notable endothermic peak at 235°C can be observed (Fig. 6f) that stems from the endothermic decomposition reaction of the abundant inorganic lithium compounds within the SEI layer [42,43], causing elevated thermal stability with a lower exothermic heat of 1.34 kJ g^−1^ of the Gr/Li hybrid anode with the MP30 electrolyte. This finding clearly indicates the improved thermal stability and dependability of the Gr/Li hybrid anode with the MP-modified EC-based electrolyte.

## DISCUSSION

In summary, the achievement of stable cycling of high-energy-density Gr/Li hybrid anodes has persistently remained challenging due to the dual issues that arise from both Gr and LMAs that are operated at high current densities, including solvent co-intercalation, dendritic-Li formation and thus low Li^+^ utilization efficiency. In addition to the facilitated desolvation process for Gr anodes, bulk ionic transport kinetics also act as the critical factor in realizing stable plating/stripping interface chemistry for Li-metal anodes. In this work, an ideal weakly solvated electrolyte has been originally designed with MP doping of a commercial EC-based electrolyte as a cosolvent, showing a loose solvation sheath via anion–dipole interaction in spite of more anion participation, in which Li^+^ moves with its flexible Li^+^–EC sheath with low EC ligancy but high ion–dipole interaction, thus demonstrating fast ion-transport capability. Characterization and simulation confirm the effect of the anion–solvent interaction within the Li^+^ solvation structure, and the rapid kinetics of desolvation and bulk ion transport are achieved simultaneously. The developed electrolyte shows remarkable cycling stability pairing with the Gr/Li hybrid anode, achieving 99.8% average CE within 1500 cycles at 4C, realizing the homogeneous and dendrite-free Li deposition and the structural integrity of the Gr. This work sheds light on a solvation design concept in traditional EC-based electrolytes and provides a unique approach to developing high-energy-density LIBs that are suitable for complex systems.

## Supplementary Material

nwaf065_Supplemental_File
