# Supplementary material for: Approaching fast ion transport via anion–dipole interaction in weakly solvated electrolytes enables stable Li-plating chemistry
Source: Natl Sci Rev. 2025 Feb 22;12(4):nwaf065. doi: 10.1093/nsr/nwaf065 (PMC11932345; doi:10.1093/nsr/nwaf065)
Supplement: nwaf065_Supplemental_File [file nwaf065_supplemental_file.pdf]

Supplementary Materials

Approaching Fast Ion Transport via Anion-Dipole Interaction in Weakly-Solvated Electrolytes Enables Stable Li Platting Chemistry

Min Niu[1], Liwei Dong[1]*, Xingyu Chen[1], Rong-Juan Feng[2], Qian Li[2], Hang Qi[1], Sen Xin[2,3], Jia-Yan Liang[1,2]*, Chunhui Yang[1]*, Yu-Guo Guo[2,3]*

1. State Key Laboratory of Space Power-Sources, School of Chemistry and Chemical Engineering, Harbin Institute of Technology, 150001, Harbin, China
2. CAS Key Laboratory of Molecular Nanostructure and Nanotechnology, CAS Research/Education Center for Excellence in Molecular Sciences, Beijing National Laboratory for Molecular Sciences, Institute of Chemistry, Chinese Academy of Sciences, 100190, Beijing, China
3. School of Chemical Sciences, University of Chinese Academy of Sciences, 100049, Beijing, China

*Corresponding author Email: dongliwei@hit.edu.cn, liangjiayan@hit.edu.cn, yangchh@hit.edu.cn, ygguo@iccas.ac.cn

MATERIALS AND METHODS

Materials

Battery-grade lithium hexafluorophosphate ($LiPF_6$), ethylene carbonate (EC), diethyl carbonate (DEC), 1,1,2,2-tetrafluoroethyl 2,2,3,3-tetrafluoropropyl ether (HFE), and high-nickel $LiNi_{0.8}Co_{0.1}Mn_{0.1}O_2$ cathode (NCM811) were purchased from Guangdong Canrd New Energy Technology Co., Ltd. The methyl propionate (MP, 99.9%) and the *N*-methyl-2-pyrrolidone (NMP, 99.0%) were purchased from Aladdin reagent. The super P and polyvinylidene fluoride (PVDF) were purchased from HF-Kejing. The spherical graphite was obtained from Heilongjiang Hagong Graphite Technology Co., Ltd. The MoO_x-MoN_x layers modified graphite anode (MoON@Gr) was synthesized with reference to our previous study (doi.org/10.1002/anie.202318663).

Electrolytes and Battery preparations

The electrolyte systems and coin-cell batteries were prepared in a glovebox under an argon atmosphere (O_2, H_2O < 0.1 ppm). The base electrolyte was prepared by dissolving 1 M $LiPF_6$ in a mixture of carbonate solvents (EC: DEC = 1:1 by volume, denoted as Base electrolyte). The test electrolytes were prepared by introducing the additional MP (10%, 30%, 50%, by volume, denoted as MP10, MP30, and MP50 electrolyte, respectively), and the additional HFE solvent (30% by volume, denoted as HFE30) into the Base electrolyte. For the fabrication of the anodic electrodes, active materials (spherical natural Gr powder or MoON@Gr powder), super P, and binder (PVDF in NMP) were mixed with a weight ratio of 92:4:4. The as-obtained slurries were stirred for 12 h, and then coated on a Cu foil. The coated Cu foil was dried at 80 ℃ in a vacuum oven for 12 h. The resulting anodic electrodes were cut into circular sheets with a diameter of 12 mm. The areal capacity of the MoON@Gr anodes was controlled to 0.2 mAh cm^{-2}. The areal capacity of the Gr anodes was controlled to 0.2, 0.6, 2.0, and 3.0 mAh cm^{-2}, respectively. The area capacity of both anodic electrodes was calculated based on the mass loading and gravimetric capacity of 370 mAh g^{-1}. The cathode was prepared by first mixing a slurry of NCM811, super P, and binder (PVDF in NMP) with a weight ratio of 90:5:5. Then, the as-obtained slurries were stirred for 12 h, and coated on an Al foil. The coated Al foil was heated under a vacuum at 80 ℃ for 12 h. The resulting cathodic electrodes were cut into a circular sheet of 10 mm in diameter. The areal capacity of the NCM811 cathode was controlled to 0.3 mAh cm^{-2} for and 0.7 mAh cm^{-2}. The area capacity of both cathodic electrodes was calculated based on the mass loading and gravimetric capacity of 200 mAh g^{-1}.

Electrolyte characteristic

FTIR spectra were acquired with a Fourier transform infrared spectrometer (Nicolet, iS50 FTIR, Thermo Scientific, KBr tablet, wave number 4000-600 cm^{-1}). The Raman spectra were obtained from a Raman spectrophotometer (HORIBA Scientific LabRAM HR Evolution) with 532.17 nm laser radiation in the range of 100-2000 cm^{-1}. The nuclear magnetic resonance (NMR) spectra were obtained using liquid spectrometer (Bruker AVIII 500WB). The diffusivity (D) is determined by the equation (1):

$$\frac{I}{I_0} = exp\left[-D(2\pi\gamma\delta G)^2\left(\Delta + \frac{4\delta}{3} + \frac{3t}{2}\right)\right] \quad (1)$$

where I/I_0 is the attenuated intensity at various gradient strengths G, γ is the gyromagnetic ratio of each nucleus, δ is the length of the gradient pulse, Δ is the time between gradient pulses, and t is the time between bipolar pulses. ^7Li NMR was used to find the diffusivity of solvated Li^+, ^{19}F was used to monitor PF_6^- anion diffusivity, and ^1H NMR was used to monitor EC solvent diffusivity. The D value can be obtained by fitting a nonlinear curve of Gaussian decay. The ionic conductivity of electrolytes and the electrochemical impedance (EIS) of Li^+ bulk transport are evaluated in the steel‖steel battery at varied temperatures from +30 to -20 ℃. Ionic conductivity is calculated by the equation (2):

$$\sigma = \frac{L}{RS} \quad (2)$$

where L represents distance of the pair of steel electrodes, the R resistance is measured by EIS and S is the contact area between steel electrode and electrolyte. The activation energy (E_a) was calculated based on the Arrhenius linear relationship between ln(σT) and $1/T$ as follows in equation (3):

$$\sigma T = A\ exp(\frac{-E_a}{kT}) \quad (3)$$

where T is the absolute temperature, A is the pre-exponential factor, and k is the Boltzmann constant (1.38×10^{-23} J K^{-1}).

To measure the Li^+ transference number (t_{Li^+}), the Li‖Li cells were assembled and a 10 mV (ΔV) direct current pulse was applied for 2000 seconds to obtain the initial currents of I_0 and I_{ss} when the current reached a steady state. The EIS test was acquired before (R_0) and after (R_{ss}) the polarization between a frequency range from 100 kHz to 100 mHz by a sinusoidal amplitude of 10 mV. The transference number was calculated by the equation (4):

$$t_{Li^+} = \frac{I_{ss}(\Delta V - I_0 R_0)}{I_0(\Delta V - I_{ss} R_{ss})} \quad (4)$$

where ΔV is the applied polarization voltage, and I_0 and I_{ss} are the initial current and steady-state current before and after polarization, respectively. Correspondingly, R_0 and R_{ss} are the initial interfacial resistance after 2000 seconds of polarization, respectively.

Material characterizations

The morphology and microstructure characteristics of the cycled electrodes were observed with field-emission scanning electron microscopy (FE-SEM, Hitachi SU8010, Japan). X-ray photoelectron spectroscopy (XPS, Thermo Fisher Scientific, K-Alpha XPS spectrometer) was employed to investigate the chemical compositions of SEI formed in the respective electrolyte. The surface roughness and 3D morphology of the cycled Gr electrodes were characterized using laser scanning confocal microscopy (Olympus DSX510). All the cycled electrodes in ex-situ characteristics were firstly disassembled in a glove box, then washed with DEC solvent several times to remove residual deposits, and finally dried in the argon-filled glove box for further tests. To investigate the thermal stability of the lithiated electrodes, the half-cells were pre-cycled for ten cycles and finally discharged to 370 mAh g^{-1}, respectively. The freezing points of electrolytes were measured under nitrogen atmosphere by the differential scanning calorimetry (DSC TA instruments, Q2000). The viscosity of electrolytes was measured by rotational viscometer (Brookfield DV3T). DSC samples for thermal stability analysis were prepared by scraping the dried electrode materials off the Cu current collector, and 1 mg of the material was sealed in a Mettler high-pressure stainless-steel pairing with 3 μL of electrolyte. The DSC measurement was conducted in DSC 214 Polyma of Netzsch from room temperature to 500 °C with a rate of 5 ℃ min^{-1}.

Electrochemical measurements

Electrochemical tests were carried out using CR2025-type coin cells with Celgard 2500 separator. For the half-cell assembly, the Gr and MoON@Gr anodes with various area capacities were used as the working electrode, while the Li metal electrode was employed as the counter electrode. The Li‖Gr half cells with various area capacity are discharged at various C rates to 370 mAh g^{-1} and charged at same rate of 1C to 2.0 V *vs.* Li^+/Li. The CR2025 full cells were assembled with NCM811 cathode and Gr anode. The N/P ratio of the NCM811‖Gr full cells with NCM811 area capacity of 0.7 mAh cm^{-2} was maintained at 1.1 and 0.9. The N/P ratio of the NCM811‖Gr full cells with NCM811 area capacity of 0.3 mAh cm^{-2} was maintained at 0.7 and 0.5. The amount of electrolyte used was 50 μL and 100 μL for Li‖Gr half cells and NCM811‖Gr full cells, respectively. Before the full cells were assembled, all the anodes were carried out in an electrochemical activation process to form a stable SEI film for 3 cycles and then harvested from Li‖Gr half cells. Then, the NCM811‖Gr full cells were assembled using these pre-activated anodes. The cycling performance tests were investigated on the battery testing system (Neware, CT-3008) with the as-obtained CR2025 cells. For the steady lithiated capacity test of the half cells, the discharging capacity was fixed with 370 mAh g^{-1} without voltage limitation, while the charging process followed galvanostatic measurement till the voltage reached 2.0 V *vs.* Li^+/Li. The full cells were galvanostatically charged and discharged in a voltage range of 2.75-4.25 V at a constant current density of 0.2C. The EIS measurements were carried out with a CHI 660C electrochemical workstation.

Computational methods

Molecular dynamics simulations of the Base electrolyte (1M $LiPF_6$ in EC: DEC = 1:1) and the MP30 electrolyte (30% MP added by volume into the Base electrolyte) were performed with the Forcite module in Materials Studio software with the COMPASS III ab initio forcefield and the Nosé thermostat. All the involved molecules were optimized firstly using Dmol3 module with BLYP functional and spin-unrestricted settings in a fine quality. Before performing simulations with the canonical ensemble (NVT) at 298K for 5 ns, the annealing process from 300k to 500k for 25 cycles was conducted, and the density of each system was equilibrated with the NPT ensemble for 1 ns. The dynamic calculations were performed with a fine quality and a time step of 1 fs and initiated with the current charges and random velocities. The trajectories were stored every 1000 fs and the last frame was employed to calculate the radial distribution probability. The average radius value ($r_{average}$) between (P)PF_6^--O(EC) species can be obtained using a weighted average by considering the distribution probability responding to each radial distance within the limited radial distance of 5 Å. Geometry optimization and electronic potential analyses within the ideal solvation structure were performed using the Dmol3 module in Materials Studio software with the generalized gradient approximation with Becke-Lee-Yang-Parr (GGA-BLYP) functional.

SUPPLEMENT NOTE S1. The details for the calculated parameters of the NCM811‖Gr/Li full cell with a low N/P ratio.

In this work, the theoretical energy density of the NCM811‖Gr full cell is calculated based on the active and inactive materials. The **Note table S1** shows the involved parameters. As the N/P ratio decreased, a portion of the reversible capacity from the Gr anode was redistributed to the LMA. This redistribution, facilitated by the high theoretical specific capacity (3860 mAh g^{-1}) of the LMA anode, resulted in reduced weight and stacked volume of the anodic electrodes (**Note table S2**). Thus, a Li-ion battery assembled with a Gr/Li hybrid anode exhibits a higher gravimetric and volumetric energy density while providing the same amount of energy compared with a normal Gr-based Li-ion battery (**Note table S3**).

Note Table S1. Materials parameters in the NCM811‖Gr full cell.

**Active Materials**	**Theoretical Discharge Capacity (mAh g^{-1})**	**Practical Discharge Capacity (mAh g^{-1})**	**Density ($g\ cm^{-3}$)**
NCM811	275	200	4.7
Graphite	372	370	2.2
Lithium Metal	3862	3862	0.53
**Inactive Cell Components**	**Thickness (μm)**	**Density ($g\ cm^{-3}$)**	**Porosity**
Binder (PVDF)	-	-	-
Carbon black	-	1.95	-
Cu foil	10	8.96	-
Al foil	13	2.7	-
Separator (Celgard 2500)	25	-	40%
Liquid electrolyte	-	1.2	-

Note Table S2. Electrodes parameters in the NCM811‖Gr full cell with low N/P ratios.

		**N/P Ratio of 1.1**	**N/P Ratio of 0.9**	**N/P Ratio of 0.7**	**N/P Ratio of 0.5**	**N/P Ratio of 0.3**
Cathode	Kind	NCM811	NCM811	NCM811	NCM811	NCM811
	Pratical discharge capacity (mAh g^{-1})	200	200	200	200	200
	Mass loading (mg cm^{-2})	25.95	25.95	25.95	25.95	25.95
	Compaction density (g cm^{-3})	3.5	3.5	3.5	3.5	3.5
	Coating thickness (μm)	70.44	70.44	70.44	70.44	70.44
	Porosity (%)	32	32	32	32	32
	Area capacity (mAh cm^{-2})	5.19	5.19	5.19	5.19	5.19
	Mass per stack area (mg cm^{-2})	25.95	25.95	25.95	25.95	25.95
	Thickness per stack area (μm cm^{-2})	70.44	70.44	70.44	70.44	70.44
Hybird anode	Kind	Gr	Gr	Gr	Gr	Gr
	Pratical discharge capacity (mAh g^{-1})	350	350	350	350	350
	Mass loading (mg cm^{-2})	16.31	13.35	10.38	7.41	4.45
	Compaction density (g cm^{-3})	1.6	1.6	1.6	1.6	1.6
	Coating thickness (μm)	93.79	76.74	59.69	42.63	25.58
	Porosity (%)	36	36	36	36	36
	Area capacity (mAh cm^{-2})	5.71	4.67	3.63	2.60	1.56
	Mass per stack area (mg cm^{-2})	16.31	13.35	10.38	7.41	4.45
	Thickness per stack area (μm cm^{-2})	93.79	76.74	59.69	42.63	25.58
	Kind	-	Li	Li	Li	Li
	Pratical discharge capacity (mAh g^{-1})	-	3862	3862	3862	3862
	Mass loading (mg cm^{-2})	-	0.27	0.54	0.81	1.08
	Thickness (μm)	-	5.07	10.14	15.21	20.28
	Area capacity (mAh cm^{-2})	-	1.04	2.08	3.11	4.15
	Mass per stack area (mg cm^{-2})	-	0.27	0.54	0.81	1.08
	Thickness per stack area (μm cm^{-2})	-	5.07	10.14	15.21	20.28
Al foil	Number	1	1	1	1	1
	Mass per stack area (mg cm^{-2})	3.2	3.2	3.2	3.2	3.2
	Thickness per stack area (μm cm^{-2})	13	13	13	13	13

Cu foil	Number	1	1	1	1	1
	Mass per stack area (mg cm^{-2})	2.89	2.89	2.89	2.89	2..89
	Thickness per stack area (μm cm^{-2})	10	10	10	10	10
Electrolyte in separator	Number	1	1	1	1	1
	Mass per stack area (mg cm^{-2})	0.77	0.77	0.77	0.77	0.77
	Thickness per stack area (μm cm^{-2})	25	25	25	25	25
	Median voltage*	3.8	3.8	3.83	3.97	4.07
	Energy per stack area (mWh cm^{-2})	19.722	19.722	19.8777	20.6043	21.12

*The median voltage of the NCM811‖Gr cells with various N/P ratios is tested by the Neware battery testing system.

Note Table S3. Mass and volume distribution in the NCM811‖Gr full cell with low N/P ratios.

Mass per stack area (mg cm^{-2})					
	**N/P Ratio of 1.1**	**N/P Ratio of 0.9**	**N/P Ratio of 0.7**	**N/P Ratio of 0.5**	**N/P Ratio of 0.3**
Cathode	25.95	25.95	25.95	25.95	25.95
Anode	16.31	13.62	10.92	8.22	5.53
Cu foil	2.89	2.89	2.89	2.89	2.89
Al foil	3.2	3.2	3.2	3.2	3.2
Carbon black and Binder	1.30	1.09	0.87	0.66	0.44
Electrolyte in seperator	12.44	10.39	8.33	6.27	4.22
Sum	62.09	57.13	52.16	47.19	42.23
Energy density (Wh kg^{-1})	317.64	345.21	381.09	436.62	500.12
**Thickness per stack area (μm cm^{-2})**					
Cathode	70.44	70.44	70.44	70.44	70.44
Anode	93.79	81.81	69.83	57.84	45.86
Cu foil	10	10	10	10	10
Al foil	13	13	13	13	13
Electrolyte in seperator	25	25	25	25	25
Sum	212.23	200.25	188.27	176.28	164.30
Energy density (Wh L^{-1})	929.27	984.87	1055.81	1168.84	1285.45

SUPPLEMENT FIGURES AND TABLES

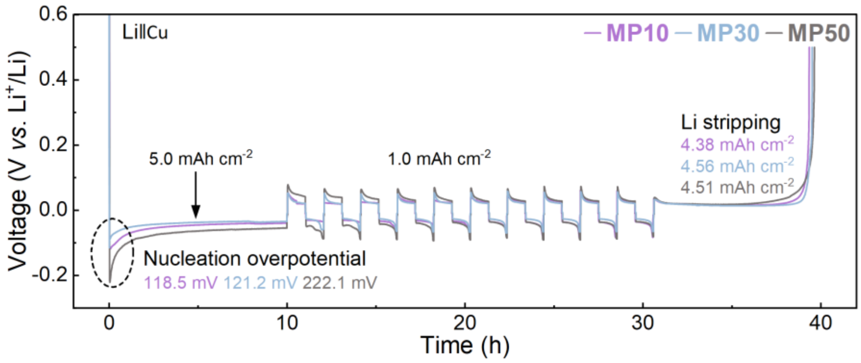

Figure S1. Li plating/stripping tests of the Li‖Cu cells with a capacity of 1.0 mAh cm^{-2} at a current density of 1.0 mA cm^{-2} in electrolytes with varying contents of the MP cosolvent.

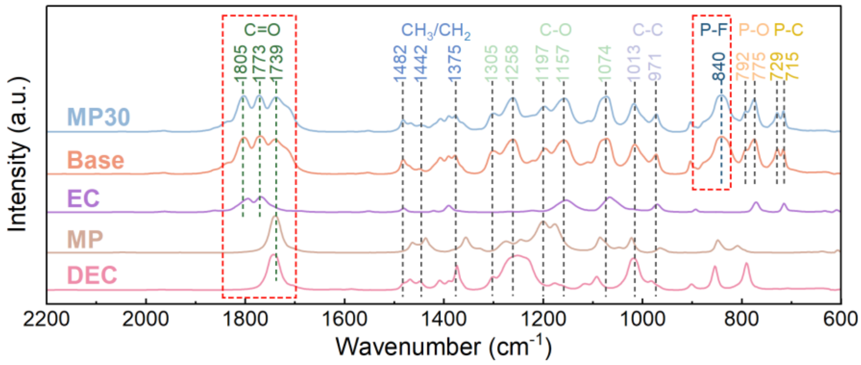

Figure S2. The ATR-FTIR spectra of EC solvent, DEC solvent, MP cosolvent, Base electrolyte, and MP30 electrolyte.

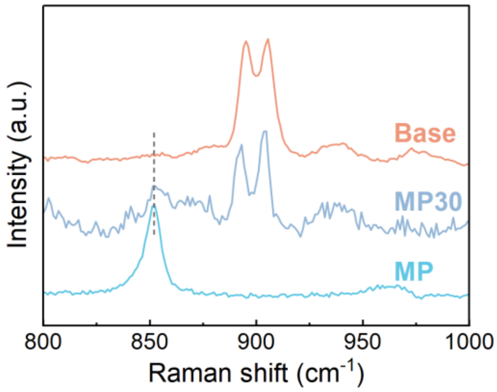

Figure S3. Raman spectra of Base and MP30 electrolytes, and the MP cosolvent.

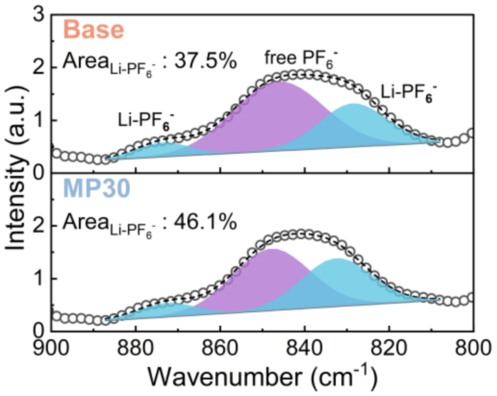

Figure S4. Fitting peaks for PF_6^- regions of the FTIR spectra in Base and MP30 electrolytes.

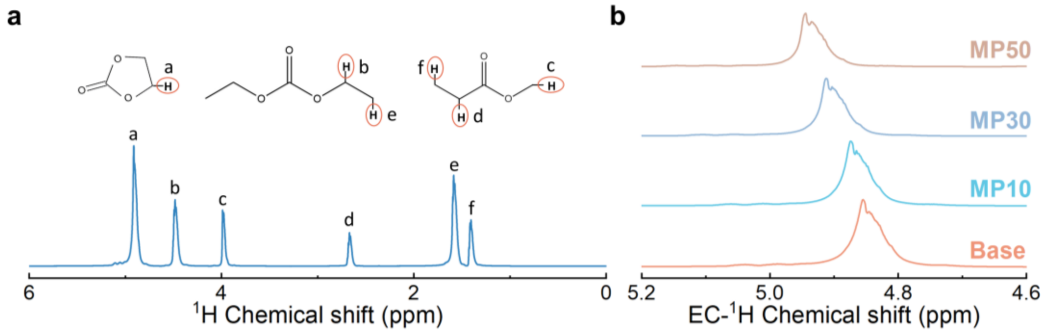

Figure S5. (a) ^{1}H NMR spectrum of EC, DEC, and MP. (b) ^{1}H NMR spectrum of EC in Base, MP10, MP30, and MP50 electrolytes.

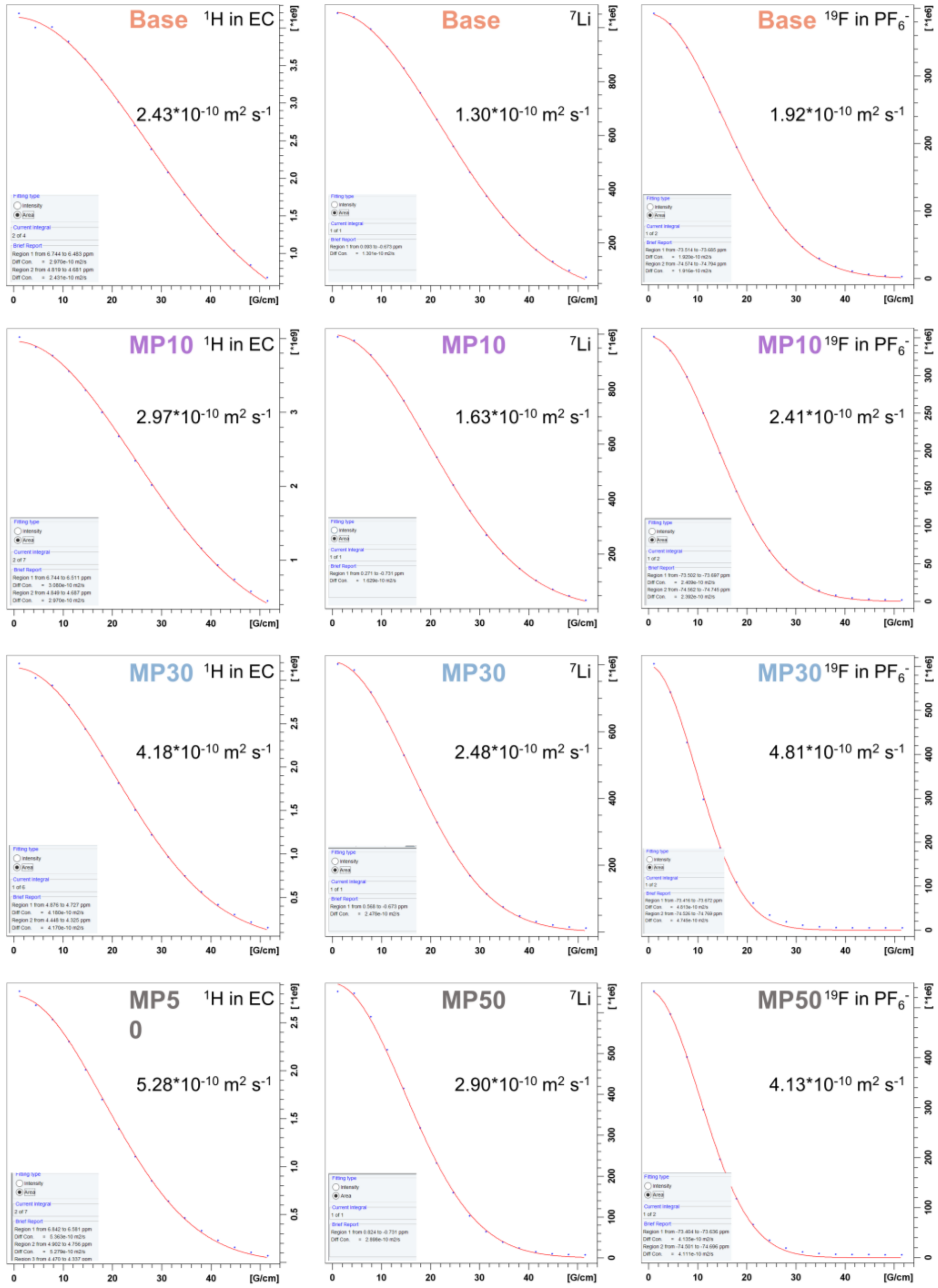

Figure S6. Fitting curves of DOSY-NMR spectra of various electrolytes.

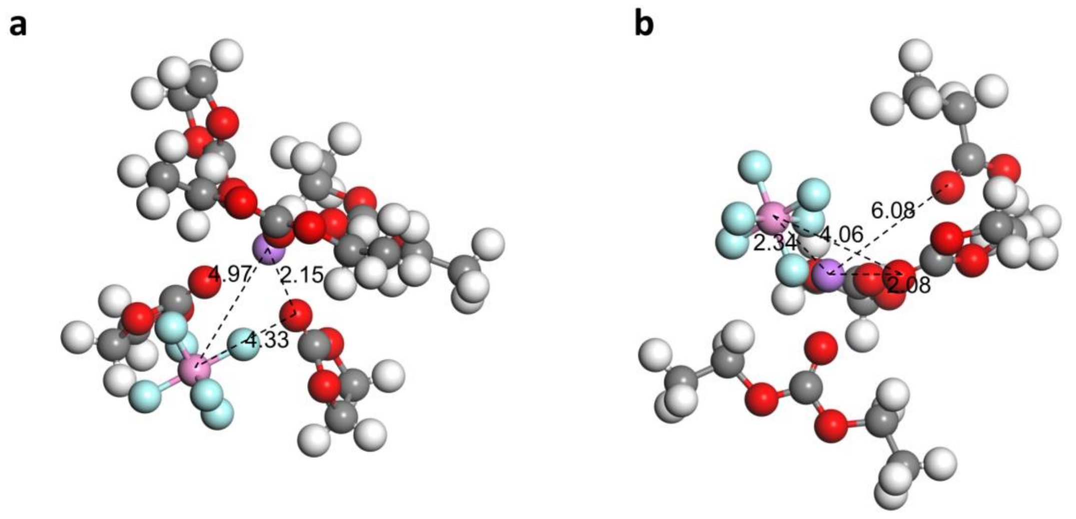

Figure S7. DFT geometry optimization of (a) Base and (b) MP30 electrolytes (Li in purple, O in red, C in gray, F in light blue, H in white, and P in pink. Rate: Å).

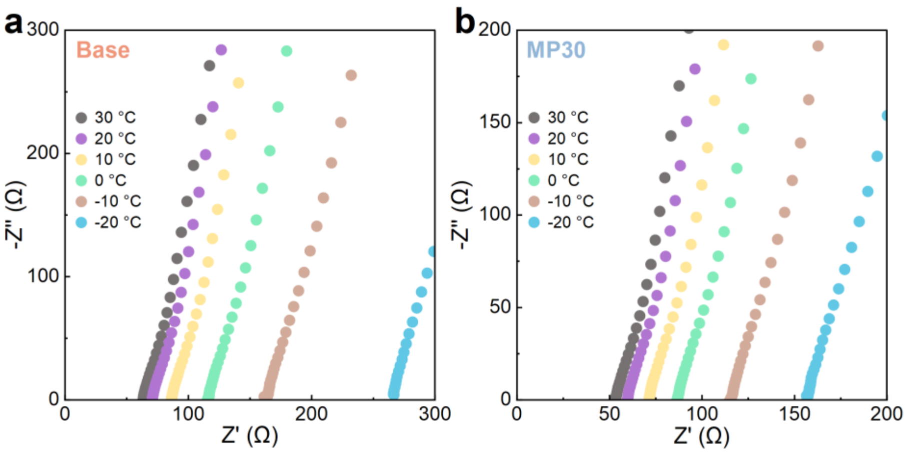

Figure S8. Temperature-dependent EIS in the range of +30 to -20 ℃ for Steel‖Steel cells containing (a) Base and (b) MP30 electrolytes.

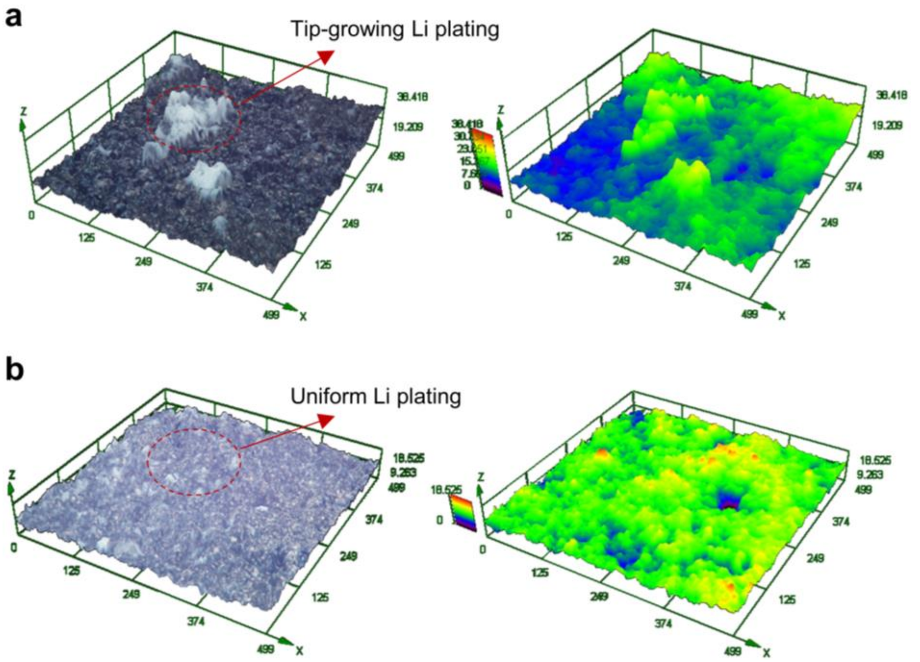

Figure S9. The 3D roughness reconstruction of the lithiated Gr anode after 30 cycles with a steady lithiated capacity of 370 mAh g^{-1} at 2C in (a) Base and (b) MP30 electrolytes.

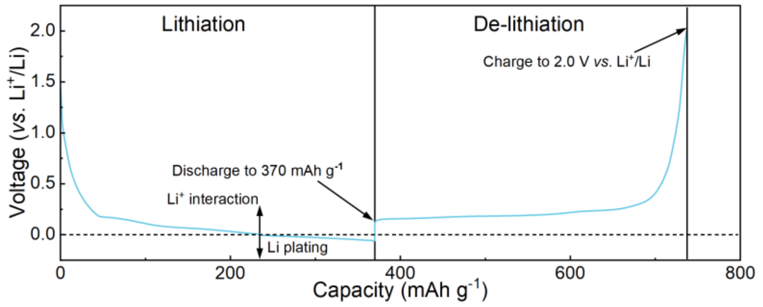

Figure S10. The steady lithiated capacity test process of the hybrid Gr/Li anode.

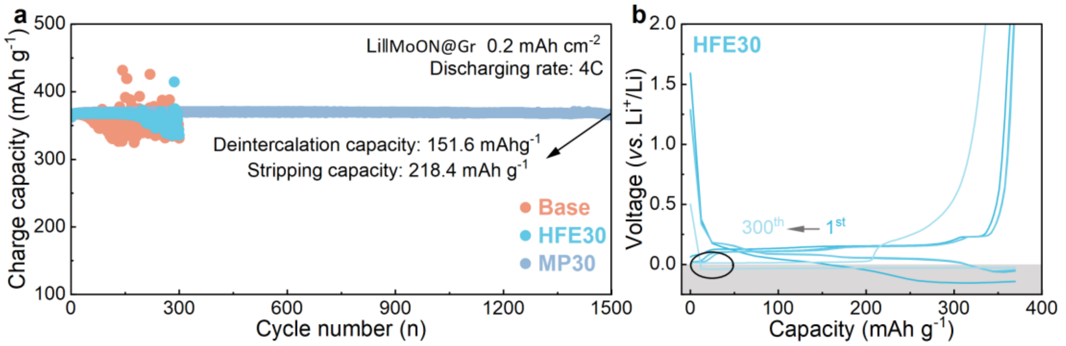

Figure S11. (a) The charge capacity of the Li‖Gr cells with Gr area capacity of 0.2 mAh cm^{-2} using different electrolytes, discharging at 4C. (b) The selected voltage curves of the Li‖Gr cell with the HFE30 electrolyte. The black-circled region exhibits polarization and overpotential as the cycle deteriorates.

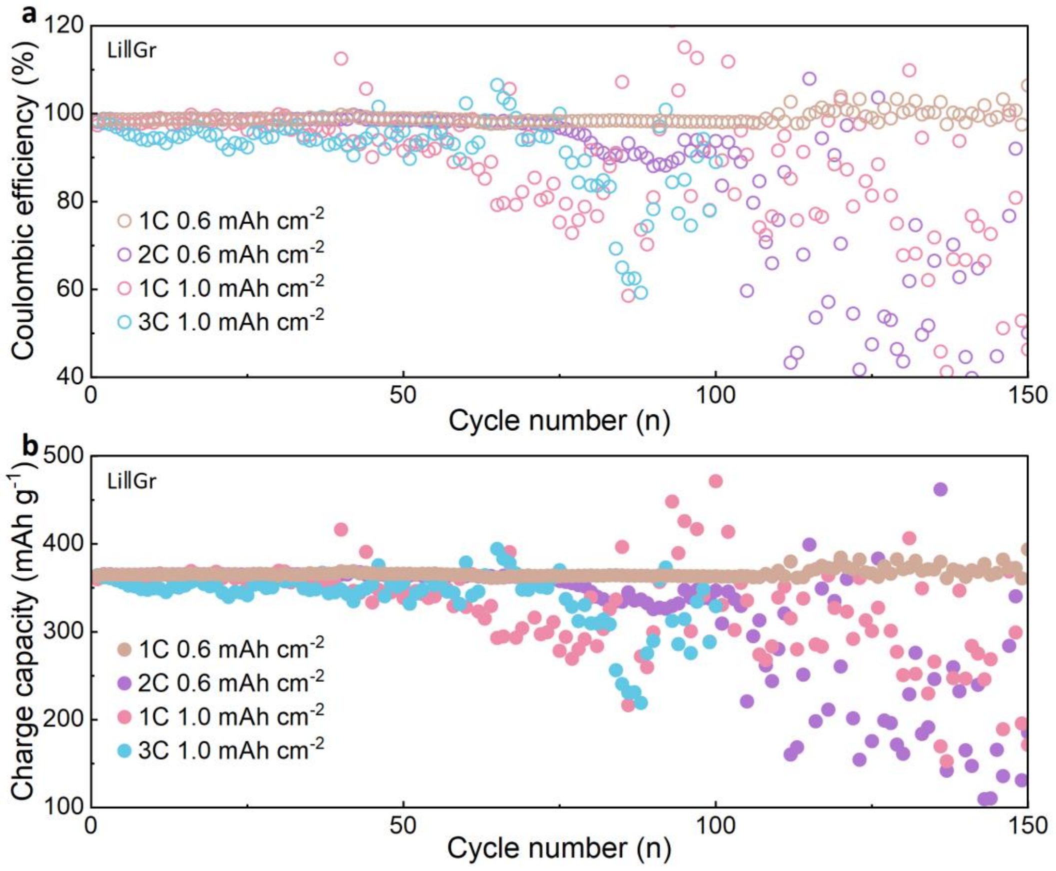

Figure S12. (a) The CE and (b) the corresponding charge capacity of Li||Gr cells cycled in the HFE30 electrolyte under different discharging rates and area capacities.

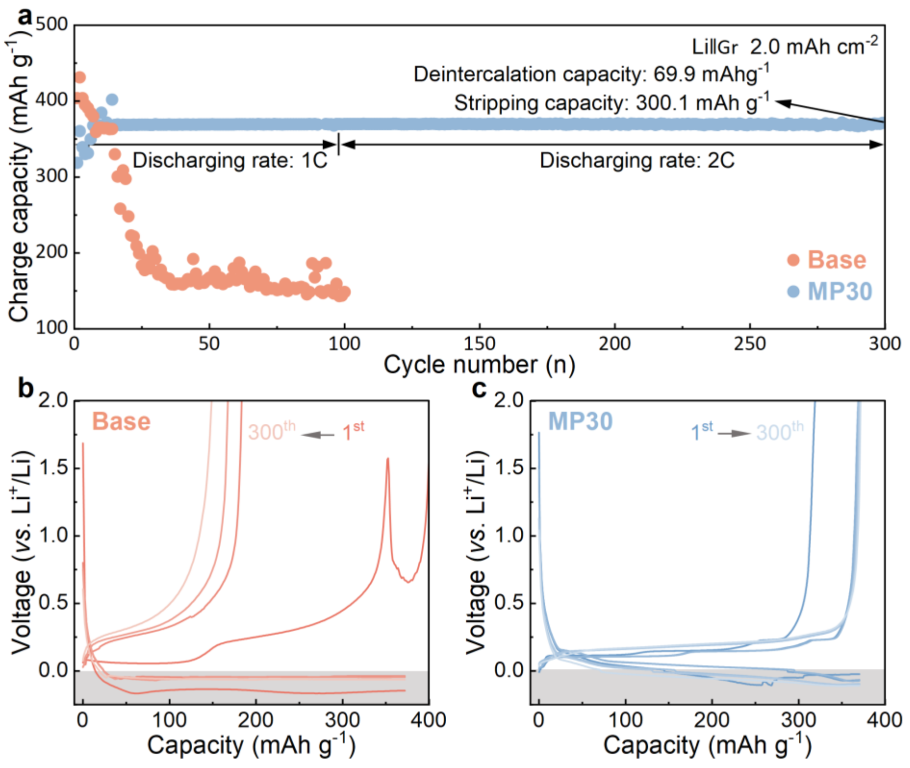

Figure S13. (a) The charge capacity of the Li||Gr cells with Gr area capacity of 2.0 mAh cm^{-2} using different electrolytes, discharging at 1C/2C. The selected voltage curves of the Li||Gr cells with (b) Base and (c) MP30 electrolytes.

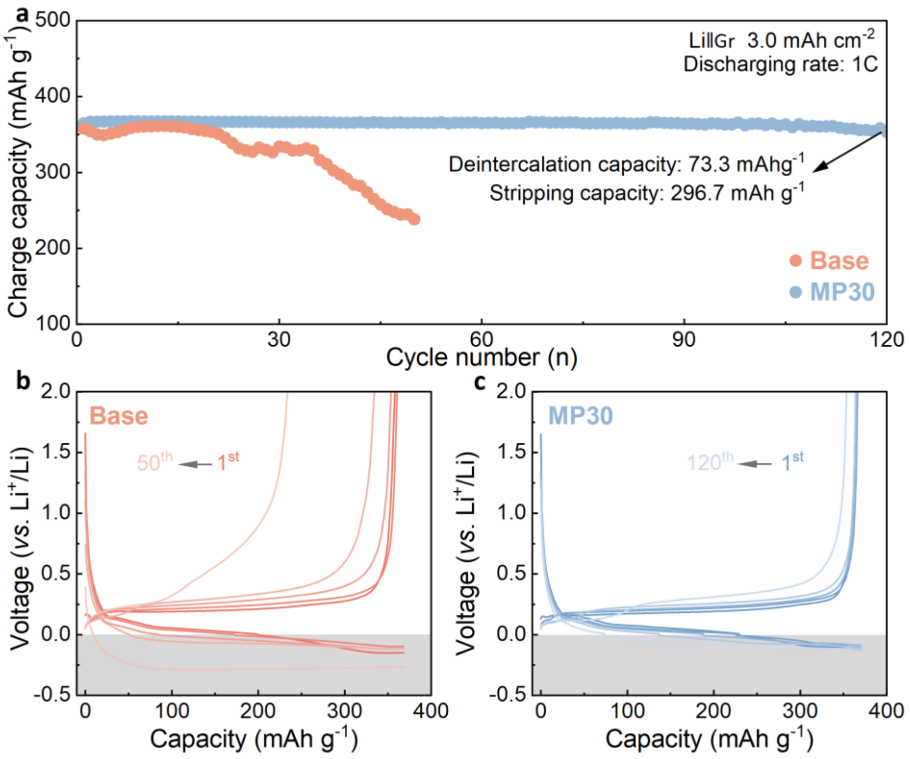

Figure S14. (a) The charging capacity of the Li‖Gr cells with Gr area capacity of 3.0 mAh cm^{-2} using different electrolytes, discharging at 1C. The selected voltage curves of the Li‖Gr cells with (b) Base and (c) MP30 electrolytes.

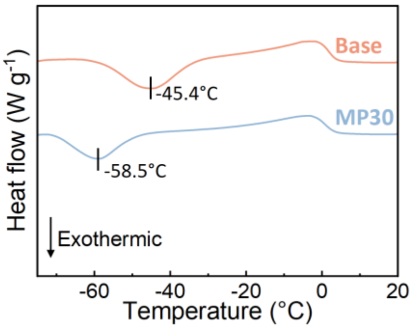

Figure S15. DSC heating curves at temperature range of -200-25℃.

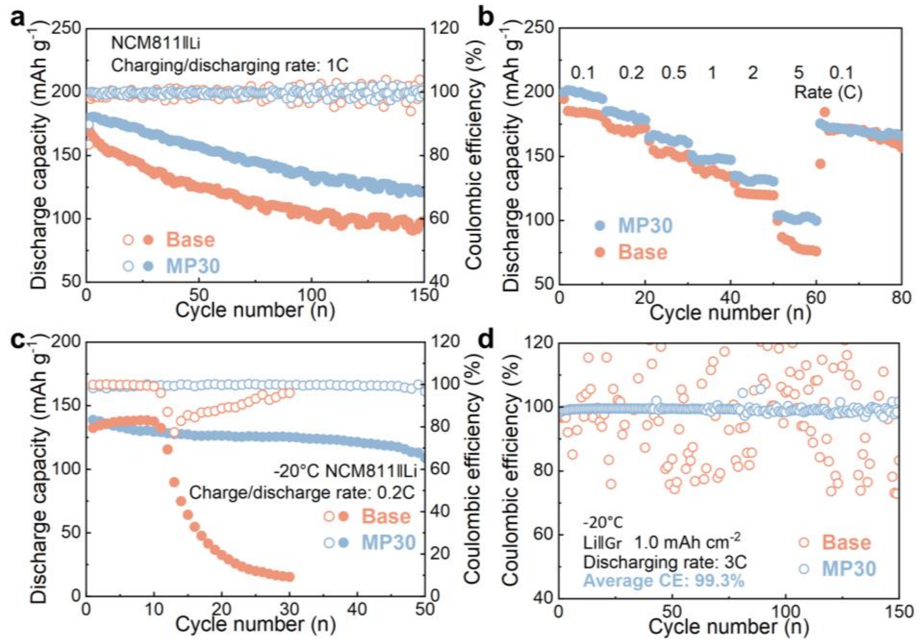

Figure S16. (a) Cycling performance and (b) rate performance of the NCM811‖Li cells with a voltage range of 2.8V-4.4V. Cycling performance of (c) NCM811‖Li at charging/discharging rate of 0.2C and (d) Li‖Gr cells with Gr area capacity of 1.0 mAh cm^{-2} at discharging rate of 3C under -20 ℃.

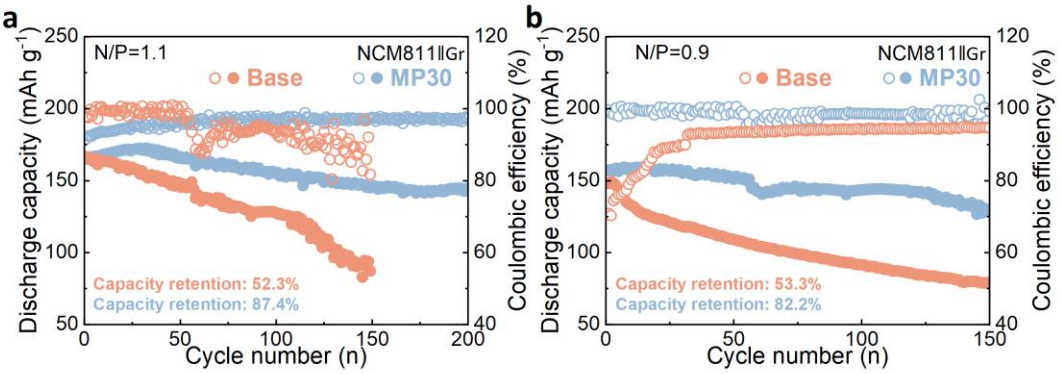

Figure S17. Cycling performance of the NCM811‖Gr full cells using different electrolytes at the N/P ratio of (a) 1.1 and (b) 0.9.

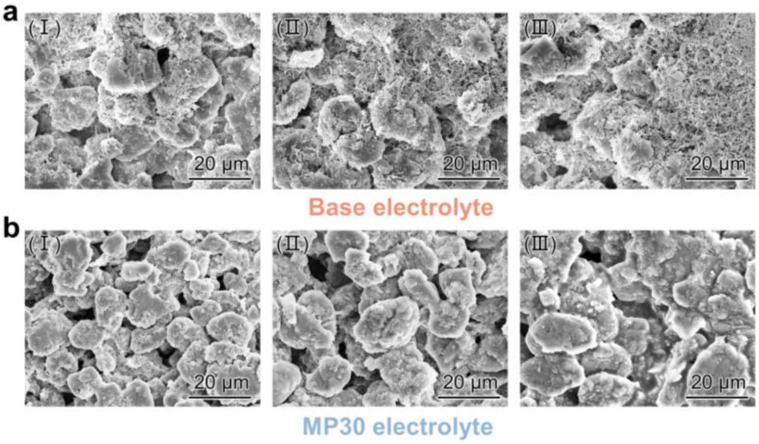

Figure S18. SEM images of the Gr electrodes during the 30th discharging process in (a) Base and (b) MP30 electrolytes.

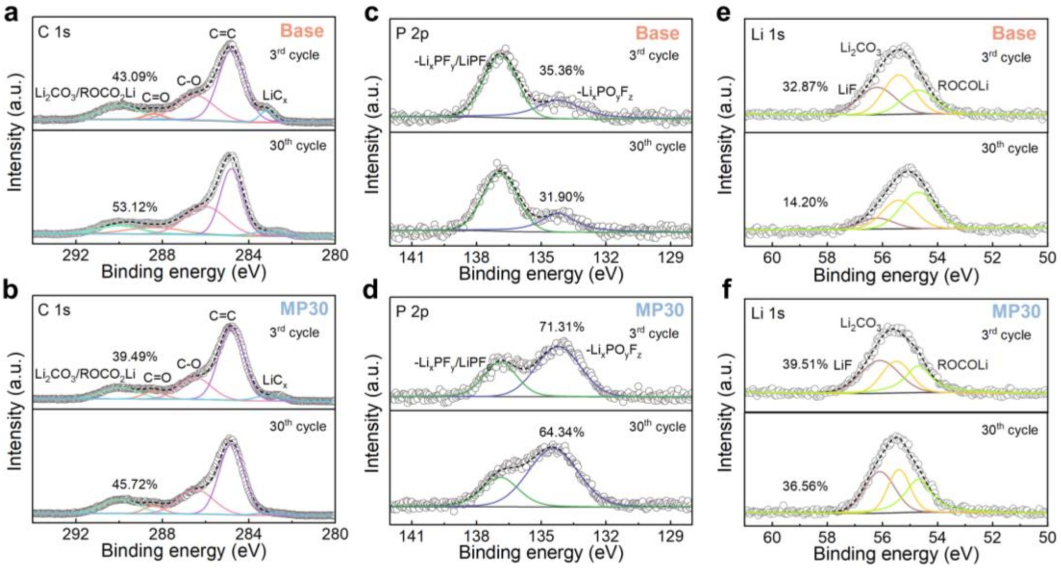

Figure S19. XPS C 1s spectra of SEI layers formed in (a) Base and (b) MP30 electrolyte, XPS P 2p spectra of SEI layers formed in (c) Base and (d) MP30 electrolyte, and XPS Li 1s spectra of SEI layers formed in (e) Base and (f) MP30 electrolyte after 3 and 30 cycles at 2C.

Table S1. Fitting parameters of the C=O vibration modes in the region of 1650-1900 cm^{-1}.

Sample	Fitting peak	Peak position (cm^{-1})	FWHM	Area percentage (%)
Base	A1	1837.6	27.4	6.4
	A2	1802.5	30.4	29.9
	A3	1769.7	26.7	22.9
	A4	1740.1	29.2	22.2
	A5	1714.4	28.9	18.6
MP30	A1	1837.4	25.3	6.2
	A2	1803.4	27.9	26.6
	A3	1771.9	20.9	17.8
	A4	1738.0	35.3	40.6
	A5	1711.7	23.1	8.8

FTIR analyses confirmed the evolution of the electrolyte solvation microstructures in Base and MP30 electrolytes. The fitting peak parameters were presented in Table S1. The ratios of solvated EC to total EC (R_1) and solvated DEC to total DEC (R_2) (calculated with equation. S1 and equation. S2) were used to quantify the relative abundance of solvated EC/DEC solvents.

$$R_1 = \frac{A_{solvated\ EC}}{A_{free\ EC} + A_{solvated\ EC}} \quad \text{(S1)}$$

$$R_2 = \frac{A_{solvated\ DEC}}{A_{free\ DEC} + A_{solvated\ DEC}} \quad \text{(S2)}$$

where $A_{solvated\ EC}$, $A_{free\ EC}$, $A_{solvated\ DEC}$ and $A_{free\ DEC}$ are the integrated area intensities of the vibrational bands corresponding to the C = O groups of solvated EC, free EC, solvated DEC and free DEC, respectively. Quantitative analysis revealed that both the R_1 and R_2 values decreased as the MP cosolvent introduction. Specifically, the R_1 value for the MP30 electrolyte decreased from 0.43 (the Base electrolyte) to 0.40, along with a decrease in R_2 from 0.45 (the Base electrolyte) to 0.18. This change clearly illustrated the weakened coordination interactions of EC/DEC. Note that the R_2 value decreased faster than R_1, resulting from the weak affinity of DEC molecule to Li^+, causing more DEC to be pushed out of PSS than EC.

Table S2. The viscosity of electrolytes.

Samples	Speed (RPM)	Torque (%)	Point	Shear Stress (dyn cm^{-1})	Shear Rate (1 S^{-1})	Viscosity (cP)
Base	100	1.7	1	7.18	132	5.44
			2	7.18	132	5.44
			3	7.18	132	5.44
MP30	100	1.4	1	5.91	132	4.48
			2	5.91	132	4.48
			3	5.91	132	4.48

Table S3. Cycling performances of the Gr/Li hybrid anode with previously reported electrolytes.

Anodic materials	Electrolytes	Discharging rate (C)	Cycle number (n)	Ref. No
Ag/HC	1.49 M LiFSI in DEM/TTE (1.2:3 by volume)	1	125	38
Graphite	1.0 M $LiPF_6$ in EC/DMC (1:1 by volume) with 5.0 vol.% FEC and 5.0 vol.% GLN	2	180	39
Graphite	1.6 M LiFSI in DMC/EC/HFE (30:1:40 by volume)	2	250	11
Graphite	1.5 M LiFSI in DME/HFE (1:2 by volume)	0.5	100	40
Graphite	1.0 M $LiPF_6$ in EC/DEC with 10 wt.% FEC and 1 wt.% VC	0.2	85	41
dCNT-G	1.3M $LiPF_6$ in EC/EMC/DEC (3:5:2 by volume) with 10% FEC, 0.2% $LiBF_4$, 0.5% VC and 1% PS	0.3	300	42
EG	1.0 M LiTFSI in DOL/DME (1:1 by volume)	1	1000	43

	with 1 wt.% $LiNO_3$			
C@Ag75	1.0M $LiPF_6$ in EC/DMC (1:1 by volume)	1	200	44
Graphite	1.4 M LiFSI in DMC/EC/BTFE (25:1:40 by volume)	0.33	100	45
Graphite	1.0 M LiFSI in EC/DEC/EMC (1:1:1 by volume)	1	560	46
Si/G	1.3 M $LiPF_6$ in EC/EMC/DEC (3:5:2 by volume) with 0.2 wt.% $LiBF_4$, 10 wt.% FEC, 0.5 wt.% VC, and 2 wt.% PVA-CN	0.5	200	47
Graphite	0.77 M $LiPF_6$ in EC/DEC (1:1 by volume) with 30 vol.% MP	4	1500	This work
